# Women’s Barriers to Weight Loss, Perception of Future Diabetes Risk and Opinions of Diet Strategies Following Gestational Diabetes: An Online Survey

**DOI:** 10.3390/ijerph17249180

**Published:** 2020-12-08

**Authors:** Kristy L. Gray, Lois McKellar, Sharleen L. O’Reilly, Peter M. Clifton, Jennifer B. Keogh

**Affiliations:** 1 UniSA: Clinical and Health Sciences, University of South Australia, Adelaide, SA 5001, Australia; kristy.gray@mymail.unisa.edu.au (K.L.G.); Lois.McKellar@unisa.edu.au (L.M.); Peter.Clifton@unisa.edu.au (P.M.C.); 2 Alliance for Research in Exercise, Nutrition and Activity (ARENA), University of South Australia, Adelaide, SA 5001, Australia; 3 School of Agriculture and Food Science, University College Dublin, Belfield, D04 V1W8 Dublin, Ireland; sharleen.oreilly@ucd.ie

**Keywords:** diabetes risk, gestational diabetes, weight loss barriers, diet strategies, intermittent energy restriction, theoretical domains framework

## Abstract

Weight loss after gestational diabetes (GDM) reduces the risk of type 2 diabetes (T2DM); however, weight loss remains challenging in this population. In order to explore perceptions of T2DM risk, barriers to weight loss, and views of diet strategies in women with previous GDM, a cross-sectional online survey of *n* = 429 women in Australia aged ≥18 years with previous GDM was conducted. Opinions of intermittent energy restriction (IER) were of interest. Seventy-five percent of responders (*n* = 322) had overweight or obesity, and 34% (*n* = 144) believed they had a high risk of developing T2DM. Within the Theoretical Domains Framework, barriers to weight loss were prominently related to Environmental Context and Resources, Beliefs about Capabilities, and Behavioural Regulation. Exercising was the most tried method of weight loss over other diet strategies (71%, *n* = 234) and weight loss support by a dietician was appealing as individual appointments (65%, *n* = 242) or an online program (54%, *n* = 200). Most women (73%, *n* = 284) had heard of IER (the “5:2 diet”), but only 12% (*n* = 34) had tried it. Open comments (*n* = 100) revealed mixed views of IER. Women in Australia with previous GDM were found to lack a self-perceived high risk of developing T2DM and expressed barriers to weight loss related to their family environment, beliefs about their capabilities and behavioural regulation. IER is appealing for some women with previous GDM; however, views vary.

## 1. Introduction

Gestational diabetes (GDM), defined as glucose intolerance that begins or is first recognized during pregnancy [1], is a growing health issue around the world. In 2016–2017, 40,000 (15%) pregnancies in Australia were affected by GDM [2]. Globally, GDM affected 16% of live births in 2019, with 20.4 million babies being born from a GDM pregnancy [3]. Although GDM typically resolves shortly after birth, the risk of developing type 2 diabetes (T2DM) is nearly 10 times higher for women with a history of GDM compared to women who did not have GDM in pregnancy [4]. Being in an overweight or obese weight category further increases the risk of developing T2DM after GDM [5,6]. A 2010 study found the overall adjusted population-attributable fraction of GDM due to overweight and obesity to be 46.2% (95% CI = 36.1, 56.3) [6].

Globally, T2DM is a major health problem, with an estimated 463 million adults in the world currently living with the disease [7]. Lifestyle modification resulting in weight loss can significantly reduce the risk of T2DM after GDM [8]; however, weight loss is not easily achieved or maintained in this population [9]. Understanding the high risk of developing T2DM associated with GDM is important for women to improve motivation to overcome barriers and make behavioural changes to prevent T2DM [10]. Exploring barriers to weight loss after GDM is also necessary to be able to implement appropriate and effective interventions [11]. Previous research investigating factors that influence lifestyle behaviour modification for diabetes prevention in women who previously had GDM identified such factors as tiredness, lack of time, lack of family support, diet not fitting into family meals, lack of affordable weight loss programs, access to child-minding services, and further pregnancies as barriers to weight loss [12,13,14]. These studies tend to target women who have given birth within the last 12 months. Although this may be the optimal time to lose weight [15], women with a history of GDM who are greater than one year postpartum may respond better to a weight loss program once a more stable lifestyle has resumed [10].

There are few large studies investigating risk perception of T2DM, barriers to weight loss, or diet strategies for women with previous GDM beyond the 12-month postpartum period. The purpose of this survey was to explore women’s perception of their risk of T2DM after GDM, to use the Theoretical Domains Framework (TDF) to identify barriers to weight loss, and to identify diet strategies, programs, or services that these women feel would be suitable to them at any time after GDM. Views and opinions of IER were of particular interest.

## 2. Materials and Methods

### 2.1. Participants and Recruitment

This study was an exploratory, cross-sectional online survey. Recruitment occurred between March and September 2018 and was done online through advertising on Facebook and Australian online parenting websites and forums, as well as a mailout (*n* = 1600) and email (*n* = 2560) by the Australian National Diabetes Services Scheme (NDSS). Facebook advertisements appeared on the newsfeed webpage of potential participants who met a predefined criterion (women, aged ≥18 years, located in Australia). The mail and email were sent by the NDSS to women on the National Gestational Diabetes Register, and included information about the survey, as well as the URL and links to access the survey and consent form. The research team did not receive the names, contact information, or any other information about anyone who clicked on the survey link from the advertisements. Since 2011, all women in Australia who are diagnosed with GDM are automatically added to the National Gestational Diabetes Register unless they opt out, and therefore, the survey advertisements reached potential participants with a diverse background. Responders were women aged ≥18 years living or residing in Australia who had been diagnosed with GDM during a pregnancy any length of time ago. Women were excluded if they had a diagnosis of type 1 diabetes (T1DM) or T2DM prior to a GDM pregnancy, or if they were pregnant with their first child (*n* = 3). Women who were currently pregnant with GDM were included if they also had GDM in a previous pregnancy. Diagnoses of GDM, T1DM, T2DM, and impaired glucose tolerance or prediabetes were self-reported. As recruitment was achieved through potential participants following a link to the survey, the research team did not have access to any medical records.

### 2.2. Survey Development

The initial survey was developed online using Survey Monkey by a dietician at the University of South Australia. The survey underwent content validation by a panel of experts in the fields of nutrition, midwifery, psychology, and health/medical research (*n* = 6). Experts responded to the clarity, coherence, relevance, and response options for each question, as well as the fit of the chosen domain within the TDF. The TDF is a theoretically-based approach to classifying attitudes and behaviours, barriers, and facilitators to behaviour change [16]. The 14 domains of the TDF can be integrated into the COM-B model (Capability, Opportunity, Motivation—Behaviour) allowing for further identification of the determinants of a behaviour [17]. The survey achieved content validation with an overall content validity index (overall CVI) of 0.91, and all questions except one received an individual CVI (I-CVI) of >0.78 for relevance (*n* = 35). Following the expert evaluation, the adapted survey was piloted in women with a history of GDM (*n* = 20) who responded to questions on the length, importance, interest, and ease to complete and understand the survey, and provided further comments. The pilot test review questions revealed an agreement percentage of ≥86% (*n* = 12) regarding the survey’s ease to complete, understand, importance, length, and interest level, and 87% of the survey questions (*n* = 27) were answered by >90% of the pilot responders. Minor changes were made based on the pilot feedback prior to the final survey being run.

### 2.3. Survey Questions

The final survey consisted of five sections: demographic information; perception of diabetes risk and views on weight loss after pregnancy; barriers to weight loss; diet strategies and services; and opinions and use of an intermittent two-day diet (the “5:2” diet). The 5:2 diet was of particular interest as this survey formed part of a larger project investigating whether an intermittent diet would be a suitable alternative to continuous energy restriction in women with previous GDM. The survey comprised of 28 questions and included single- and multiple-choice questions and Likert scales. Several questions allowed space for optional open-text comments. The level of readability was determined to be at Grade 10 reading level (15 to 16 years old) using the Gobbledygook formula [18]. Skip logic was used at several points throughout the survey so that women were not burdened with questions that were not relevant to them. The question regarding diet strategies asked responders which (if any) diet strategies they had tried from a provided list of 12 weight loss methods, and if they had tried an approach, how well that approach worked for them. No explanation for the individual diet strategies was provided based on their wide use within this population. The question assessed how many people had tried the different diet strategies listed, and did not ask participants to rate or provide views of the different strategies. The questions regarding the 5:2 diet at the end of the survey did include information about what the 5:2 diet is and what it involves, as this was assessing participants’ views and opinions of the diet.

### 2.4. Statistics

A sample size of *n* = 384 was required to provide results that are indicative of the population of women in Australia who have had GDM with 95% confidence level and a 5% margin of error [19]. The number of 384 was based on the Australian Bureau of Statistics (ABS) calculator using a population size of 40,000. The default response rate of 50% was used.

Data were analyzed using IBM SPSS Statistical Software version 26 for Windows (IBM, Chicago IL, USA). Data were tested for normality using Q-Q plots, histograms, and Kolmogorov–Smirnov tests. Variables were not normally distributed and remained skewed after log transformation, and therefore were analyzed using Kruskal–Wallis non-parametric tests. Pearson’s chi squared test was used for categorical variables. A binomial logistic regression was run to ascertain if there were any effects of age, family history of diabetes, and body mass index (BMI) on self-reported diagnoses of T2DM or prediabetes following GDM. Significance was set at *p* < 0.05. Descriptive data are presented as the % of respondents (total number of respondents) for categorical variables and the median, and IQR for continuous variables. Results from 5-point Likert scales were collated to a three-point scale by grouping “Agree” and “Strongly Agree” responses and “Disagree and Strongly Disagree” responses together. Questions and comments from the “barriers to weight loss” section were categorized into domains of the TDF within the COM-B model using a double-coding method by two of the authors (KG and SOR). Supplementary Table S1 shows the TDF domains integrated into the COM-B model. Comments received regarding the diet strategies attempted and thoughts on the 5:2 diet were categorized into common themes arising from the comments. Body mass index (BMI) was calculated from self-reported height and weight. Responders’ answers were included in the analysis for as many questions as they had valid answers for. Women who were pregnant (*n* = 20) were excluded from the weight question and BMI calculation, as well as the question, “Do you consider yourself to be in a healthy weight range?”. Australian postcode data were used to determine Socio-Economic Indexes for Areas (SEIFA).

### 2.5. Ethics

The research was conducted according to the National Health and Medical Research Council’s (NHMRC) National Statement on Ethical Conduct in Human Research. Ethics approval was granted by The University of South Australia’s Human Research Ethics Committee prior to the project commencing (protocol #200231). Responders provided informed consent electronically before commencing the survey.

## 3. Results

Four hundred and thirty-two women began the survey. Three women were excluded as they were pregnant with their first child. There were 429 valid responses, with 77% (*n* = 329) completing the entire survey. The overall response rate could not be calculated as there were no data available on how many people saw the online advertisements; however, *n* = 4160 invitations were sent through mail and email. The median, IQR age was 36.0, 8.0 years, respectively, and BMI was 30.0, 9.8 kg/m^2^. Seventy-five percent of women (*n* = 322) were above the healthy BMI range of 18.5–24.9kg/m^2^, and five women were underweight (1%). Women were 3.0, 5.0 years post-GDM (2.4, 3.6 years postpartum). Table 1 shows the personal and demographic characteristics of the responders.

Just over 50% of women had lost the weight they gained during pregnancy (*n* = 217). Of the women who were at least 12 months postpartum (*n* = 330), 53% (*n* = 175) had lost all the weight they gained during pregnancy. Two-thirds of respondents breastfed their baby after GDM for more than three months (67%, *n* = 285) and one-third (34%, *n* = 147) breastfed for more than 12 months. Length of time breastfeeding was not associated with losing pregnancy weight (*p* = 0.14, *n* = 423).

Six women were diagnosed with T2DM since having GDM, and 10% (*n* = 43) were told they had pre-diabetes or impaired glucose tolerance (IGT). Ninety-five responders (22%) answered that they had not been tested for diabetes since having GDM. Of the responders who had not been tested for T2DM, 19% (*n* = 18) were either in an underweight or healthy weight range. Eleven women had not seen a healthcare professional since having GDM, and 38% (*n* = 164) had been told by a healthcare professional that they needed to lose weight. In a binomial logistic regression, family history of diabetes and age were significantly related to a self-reported diagnosis of prediabetes or T2DM after GDM; however, BMI was not (*p* ≤ 0.001, r^2^ = 0.167 for the model).

### 3.1. Knowledge of Weight, Weight Loss, and Perception of Risk of T2DM

Women who were in an overweight (*n* = 115) or obese (*n* = 207) weight category mostly knew that they were overweight. Only eight women who had overweight or obesity answered that they thought they were a healthy weight, and one responder with an overweight BMI identified as underweight. Women with a higher BMI were more likely to respond that they thought they were very overweight (*p* < 0.001, *n* = 413).

One-third of women (34%, *n* = 144) thought they were at high risk of developing T2DM, 41% (*n* = 176) answered moderate risk, 20% (*n* = 86) answered low risk, and 5% (*n* = 22) answered that they did not think they were at risk for developing T2DM. Higher BMI was associated with a higher perceived risk of developing T2DM (*p* < 0.001, *n* = 412). There was a statistically significant association between family history of GDM and higher perceived risk of developing T2DM (*p* < 0.001). GDM management method was also significantly associated with perceived risk of T2DM with 44% (*n* = 60) of responders who took insulin during a GDM pregnancy responding they thought they had a high risk of developing T2DM, compared to 36% (*n* = 16) of responders who took metformin and 27% (*n* = 67) who were diet-controlled (*p* = 0.008).

Eighty-three percent (*n* = 358) reported that they had thought about or tried losing weight since having GDM. Of the 65 women who had not thought about or tried losing weight since having GDM, 62% (*n* = 40) were in an underweight or healthy weight range.

Around three-quarters of responders (74%, *n* = 318) believed that gradual weight loss while breastfeeding was safe, 4% (*n* = 19) answered that you should not lose weight while breastfeeding, and 12% (*n* = 50) answered they did not know if it was safe to lose weight while breastfeeding.

### 3.2. Barriers and Enablers to Weight Loss after GDM

**Figure 1:**
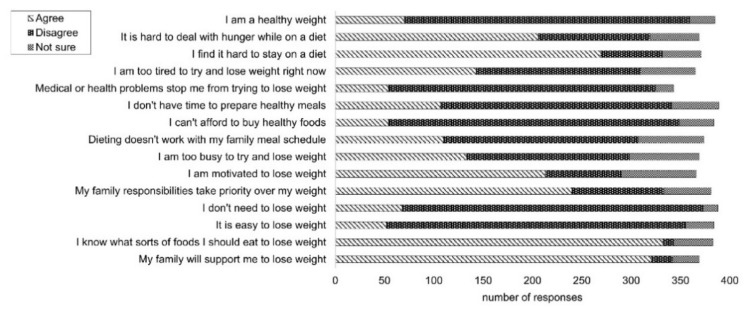
Barriers and enablers to weight loss. Data *n* = 389 responses for each statement listed. Responses were received as a 5-point Likert scale (strongly agree, agree, not sure, disagree and strongly disagree). Responses for “strongly agree” and “agree” have been grouped together as “agree”, and responses for “strongly disagree” and “disagree” have been grouped together as “disagree”. Responses for “not applicable” are not shown.

The most common barriers to weight loss were family responsibilities taking priority (63%, *n* = 240) (TDF domain: Environmental Context and Resources) and finding it hard to deal with hunger (56%, *n* = 206) (TDF domain: Behavioural Regulation). Most women agreed or strongly agreed that they knew what sorts of foods they should eat to lose weight (86%, *n* = 335) (TDF domain: Knowledge) and that their family would support them to lose weight (82%, *n* = 321) (TDF domain: Social Influences). Figure 1 shows the levels of agreement to each statement regarding barriers to weight loss. Responders who were postpartum longer were more likely to strongly disagree with the statement, “It is hard to deal with hunger while losing weight” (*p* = 0.03, *n* = 389) (TDF domain: Emotion) and “I don’t need to lose weight” (*p* = 0.02, *n* = 389). There were no other significant associations with barriers to weight loss and years postpartum.

**Figure 2:**
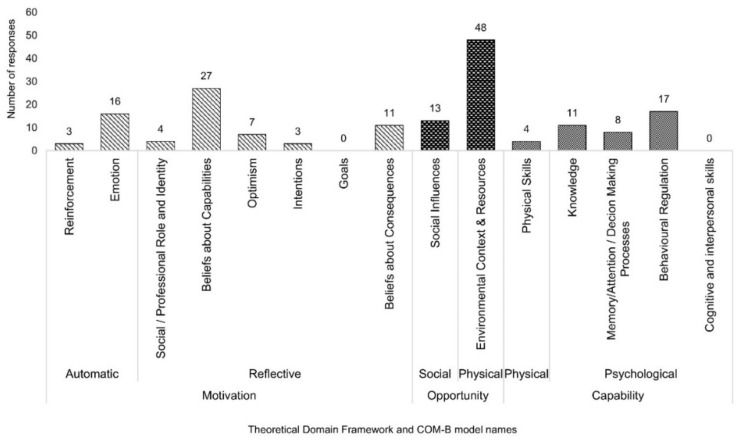
Barriers to weight loss categorized into the Theoretical Domains Framework and the COM-B (Capability, Opportunity, Motivation- Behaviour) model from responders’ comments.

There were 114 comments received in the open-text comment box regarding barriers to weight loss, which resulted in 172 phrases being categorized within the TDF. Environmental Context and Resources, and Beliefs about Capabilities were the most prominent domains within the TDF, with *n* = 48 and *n* = 27 comments sitting in the respective domains. Figure 2 shows the frequencies of each domain within the TDF categorized for this question. Supplementary Table S2 highlights some of these comments and the domain they were categorized into.

### 3.3. Diet Strategies

**Figure 3:**
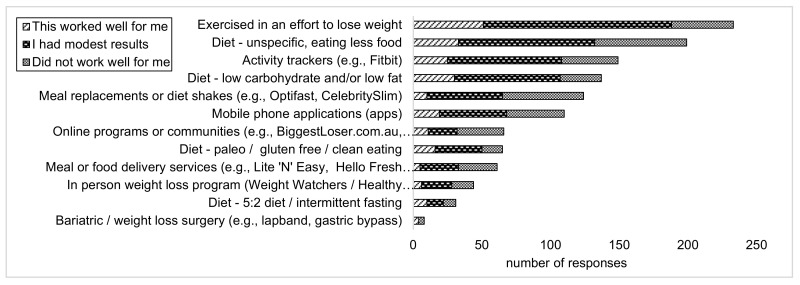
Diet strategies tried and how well they worked.

Exercising to lose weight was the most tried method of dieting with 71% (*n* = 234) of women reporting they had tried this method, and only 14% (*n* = 45) responded that exercising did not work for them. A nonspecific diet (eating less food overall) was the second most tried method of dieting, with 61% (*n* = 201) of women reporting they had tried this, and only 20% (*n* = 67) reporting that this did not work for them. Eighteen percent (*n* = 60) had used an online program to lose weight and 33% (*n* = 109) had used a mobile application. Figure 3 shows results for the types of diets and weight loss strategies tried and how well they worked for the responders.

Age was positively associated with the use of weight loss center programs. There was no significant difference in the age for responders who had or had not tried meal replacements, meal delivery services, online programs, mobile applications, bariatric surgery, low-carbohydrate or low-fat diets, intermittent fasting, paleo or gluten-free diets, eating less food, exercising, or using an activity tracker.

Other approaches that women added in the open-text comments (*n* = 64) included: the Mediterranean diet, vegan diet, avoiding processed foods, prescription weight-loss medication, “quitting sugar”, limiting sweetened beverages such as soda, self-hypnosis, psychological support, listening to podcasts, maintaining dietary changes made in GDM pregnancy, breastfeeding, keeping a food diary, and paid diet programs.

### 3.4. Weight Loss Services, Programs, and Information

Responders were asked what kind of weight loss program or service they thought would work for them. Services delivered by a nutritionist or dietician were the most popular choices, either as individual appointments (62% (*n* = 242) agreed or strongly agreed) or within an online program (51%, *n* = 200 agreed or strongly agreed). Group sessions run by a dietician or nutritionist were less popular (38%, *n* = 146 agreed or strongly agreed). Mobile applications were also a popular choice, with 50% (*n* = 194) agreeing this would be a good option for them. There was a statistically significant difference in years postpartum across the levels of agreement for group sessions with a dietician, with responders who were further post-pregnancy more likely to disagree that group sessions would work for them (*p* = 0.002, *n* = 389). These responders were also less likely to agree that an online community with other mothers (*p* = 0.03, *n* = 389) or using a mobile application (*p* = 0.001, *n* = 389) for weight loss would be helpful.

Responders were asked where they have or would go to look for information on how to lose weight. Internet searches were the most popular choice for seeking information to lose weight (*n* = 308) followed by a doctor, dietician, or nutritionist (*n* = 234), online forums or online communities (*n* = 167), family or friends (*n* = 163), diet books (*n* = 137), gym instructor/lifestyle coach (*n* = 129), television shows (*n* = 45), midwife or nurse (*n* = 41), and a psychologist or counsellor (*n* = 24).

### 3.5. Intermittent Fasting (the 5:2 Diet)

Seventy-three percent of responders (*n* = 284) had heard of the 5:2 diet, but only *n* = 34 had tried the diet (*n* = 27 tried it and lost weight, *n* = 7 answered that they tried it and it was not right for them) and 26% (*n* = 73) had thought about the diet but had not yet tried it. Responders who had not heard of the 5:2 diet were given information explaining the diet. Of the responders who had not heard of the 5:2 diet and thought they needed to lose weight (*n* = 87), 60% (*n* = 52) answered that it sounded like it could be a good option for them, 18% (*n* = 16) answered that the diet would not suit them, and 22% (*n* = 19) were unsure. Common themes that emerged from comments regarding the 5:2 diet (*n* = 100) included concerns around how the fasting days would fit in with family routines and the influence or messages this may portray to their children, as well as concerns about feeling too hungry and overeating on non-fasting days. Several women commented that they thought the diet sounded interesting or appealing but would need support and advice on how to do it, as well as meal ideas. Other women commented that they have found the diet easy and/or achievable or have heard positive stories from family and friends about the diet (Supplementary Table S3).

## 4. Discussion

Enabling lifestyle behaviour changes is a complex process [20]. One of the main findings in this survey was that although responders had good knowledge of their weight status, this did not translate to a high level of perceived T2DM risk. These findings add to the body of evidence that most women with previous GDM lack an understanding of the high risk of T2DM development [21]. Understanding the high risk of T2DM after GDM, particularly for women with overweight or obesity, is important as it offers motivation to lose weight and implement changes in behaviour to reduce the risk of diabetes [22,23]. Under the TDF and COM-B model, the question regarding perception of T2DM risk in this survey is related to an individual’s belief or acceptance of the significance of developing T2DM. Motivation to change behaviour depends on the value attached to the consequences of one’s behaviour [24], suggesting that responders may not believe that the consequence of developing T2DM is important enough to motivate the individual to change their behaviour. Other studies have also shown that women with previous GDM who held a higher perceived risk of developing T2DM were more likely to change their behaviors [22]. The cumulative incidence of T2DM after GDM is 9% within five years [4], which may not be perceived as very high by the responders. Only six responders in our study reported to have developed T2DM since GDM, and 10% (*n* = 43) had been diagnosed with prediabetes. Most responders had their GDM managed through lifestyle modification rather than medication, and this was associated with a lower perceived risk of T2DM. The lack of a high perceived risk of developing T2DM could also be in part due to most responders reporting to have received normal results from their most recent diabetes screening test, which could lessen their notion of risk. However, close to one quarter of the responders in this survey had not been tested for diabetes following their GDM pregnancy. Low levels of diabetes screening in this population are well-recognized [25], highlighting a missed opportunity for diagnosis and early intervention.

Understanding the barriers to weight loss that women with previous GDM may experience can help to guide the development of appropriate and effective interventions. Results from this survey suggest that the Environmental Context and Resources, Behavioural Regulation, and Beliefs about Capabilities are key domains of the TDF regarding weight loss barriers that women with previous GDM may experience. Using the COM-B model, this highlights a perceived lack of opportunities and a lack of motivation to make lifestyle changes. Many of the comments categorized to the Environmental Context and Resources domain relate to family responsibilities, with factors such as needing to cook separate meals for children and/or partners, not having time to exercise, and breastfeeding frequently being cited in this domain. Medical issues were also commonly mentioned. The barriers to weight loss identified in this study are not unique to women with previous GDM. Family responsibilities, tiredness, and self-regulation issues are also commonly cited as barriers to weight loss in a general population of postpartum women with overweight or obesity [26]. Responders in this survey who were further postpartum showed better self-regulation in dealing with hunger than responders who had recently had a baby. These findings are consistent with recent evidence which shows that women who are less than two years postpartum benefit from lifestyle modification that includes strategies such as self-monitoring to improve behavioural regulation, setting and reviewing goals, and problem solving, rather than information provision to increase knowledge [27].

Exercising for weight loss was the most tried approach to lose weight by the responders in this survey. Exercising alone for weight loss requires no change in dietary behaviors. Given the difficulties women mentioned around changing dietary behaviors with a young family at home, this could be one reason why women are choosing exercise for weight loss. While exercise improves body composition and holds an important role in the prevention of T2DM, weight loss from exercise alone is typically small, and energy restriction through dietary changes is also required to achieve clinically significant weight loss [15,28].

In recent years, alternative weight loss strategies, such as intermittent energy restriction (IER) have become popular in general populations. There are many forms of IER, with one of the most common being the “5:2 diet”, requiring severe energy restriction for two days per week and habitual eating for five days per week [29]. This is appealing to some, given it only requires a behavioural change for two days each week, therefore creating less disruption to daily life [30]. Given the barriers to weight loss that women in this study have identified, specifically factors relating to lack of time and family responsibilities, as well as a lack of motivation to change, a two-day-a-week diet may be an achievable weight loss strategy for some women with previous GDM. The results from this survey presented equivocal views and opinions of the 5:2 diet, including some women who thought the diet was an excellent approach, and others who strongly felt this would not be a good diet for them, reinforcing that there is no single approach that will work for everyone. A qualitative study of women’s experiences following the 5:2 diet for reduced risk of breast cancer reported positive psychological results, with many participants finding the 5:2 diet easier to adhere to and less demanding than a continuous diet, and that it increased their motivation and self-efficacy [31]. However, the women in this study were in a different life stage to the responders in the current study and may not experience the same barriers to weight loss related to caring for a young family. Further research investigating the qualitative aspects of this diet approach in women with previous GDM or postpartum women would be beneficial. Additionally, over 200 participants in the survey reported dealing with hunger as a barrier to weight loss, which needs to be considered for people commencing a 5:2 diet, given the severe energy restriction on fasting days. However, other research has found that hunger is not increased on an intermittent diet, can produce weight loss and metabolic benefits comparable to a continuous diet, and is a suitable alternative weight loss strategy to a continuous diet [32,33].

## 5. Limitations

There were several limitations in this study. One of the aims of this study was to explore women’s perceptions of their risk of developing T2DM after GDM. Participants were offered four levels of risk to choose from (high, moderate, low risk, or not at risk). However, the perception of level of risk is somewhat subjective, and we did not provide an explanation of what high-, medium-, or low-risk meant. However, our results were consistent with other research in the area showing that women with previous GDM do not perceive themselves to be at high risk for developing T2DM. Future research could use focus groups to gather a deeper understanding of perceived level of risk; however, this is a trade-off for achieving the large sample size that we did. The second aim of our survey was to use the TDF to identify barriers to weight loss at any time after GDM. The limitation of this aim was that the TDF question items were not equally spread in number for each TDF domain, and therefore domain ranking could not be performed. However, the barriers to weight loss responses showed Environmental Context and Resources, Beliefs about Capabilities, and Behavioural Regulation as key domains. We did encourage free text comments in this question and linked these to the TDF, which allowed us to see which domains in the TDF participants expressed barriers to weight loss in addition to the provided list. Further research investigating barriers to weight loss after GDM should include an equal number of barriers assigned to each domain to allow for domain ranking. Our sample corresponds to approximately 1% of the 40,000 women who gave birth in Australia in 2016–2017 diagnosed with GDM. We recognize this was a small sample size and may not represent the views of the overall population with GDM in Australia. However, we believe it provides insight into the perception of future diabetes risk in this group of women, and a larger survey would help clarify this. This survey aimed to reach a diverse population of women with previous gestational diabetes by including a letter and email sent out for recruitment by the NDSS to women on the Gestational Diabetes Register. Although we cannot be sure how many participants participated in the survey after receiving the letter or email and how many participated after seeing an advertisement on Facebook or an online parenting website, the SEIFA index, which ranks the level of disadvantage based on postcode data in deciles, showed participants were predominantly living in a middle-of-the-range socio-economic area, with a large IQR and with a good spread of diversity across the SEIFA deciles. Another consideration was the lack of eligibility criteria set for weight or BMI in this study. As weight was self-reported in this study and women who are overweight commonly underestimate their weight [34], it was useful to include women who may not know that they are overweight. Further to this, as this survey investigated opinions of the effectiveness of different diet strategies, it was valuable to include the experiences from women who may have previously been overweight and were now classified into a healthy weight range.

## 6. Conclusions

This large Australian survey found that, overall, women with previous GDM do not perceive themselves to have a high level of risk for developing T2DM. Diabetes prevention interventions need to consider the restrictions to lifestyle changes imposed on women with a young family and facilitate improving self-regulation and motivation. The 5:2 diet may be an achievable option for some women with previous GDM who are above a healthy weight range; however, further research in this area is needed.

## Figures and Tables

**Table 1:** Characteristics of survey responders.

Characteristic	All Responders
Age, y	36.0, 8.0
Weight, kg	82, 27
BMI, kg/m^2^	30.1, 9.7
SEIFA *, index	6.0, 5.0
- Decile 1, *n* (%) - Decile 2, *n* (%) - Decile 3, *n* (%) - Decile 4, *n* (%) - Decile 5, *n* (%) - Decile 6, *n* (%) - Decile 7, *n* (%) - Decile 8, *n* (%) - Decile 9, *n* (%) - Decile 10, *n* (%)	43 (10) 27 (6) 53 (13) 40 (10) 38 (9) 54 (13) 41 (10) 63 (15) 34 (8) 29 (7)
Years postpartum ** y	2.5, 3.5
Years since GDM, y	3.0, 5.0
GDM managed, *n* (%)	
- Diet/Exercise - Metformin - Insulin	248 (7) 46 (11) 135 (31)
Children living at home, n	2, 1
Employment, *n* (%)	
- Full-time work/study - Part-time work/study - Stay-at-home /maternity leave - Retired/unemployed/other	99 (23) 180 (42) 126 (29) 24 (5)
Family history of diabetes, *n (%)*	268 (62)
Lost weight gained in pregnancy, *n* (%)	
- Yes - No - “I don’t know”	218 (62) 177 (42) 30 (7)
Breastfeeding time, *n* (%)	
- Did not breastfeed - Breastfed up to 3 months - Breastfed 4–6 months - Breastfed 1 year or longer	40 (9) 99 (24) 46 (11) 147 (35)

Data is median, IQR. * Socio-Economic Indexes for Areas (decile 1 = most disadvantaged, decile 10 = least disadvantaged), ** from most recent pregnancy.

## Supplementary Materials

The following are available online at https://www.mdpi.com/1660-4601/17/24/9180/s1, Table S1: The Theoretical Domains Framework integrated into the COM-B model (adapted from Cane et al 2012, pg. 15), Table S2: Responder comments on barriers to weight loss mapped to the Theoretical Domains Framework, Table S3: Responder comments regarding opinions of the ‘5:2 diet’.
