# Supplementary material for: Women’s Barriers to Weight Loss, Perception of Future Diabetes Risk and Opinions of Diet Strategies Following Gestational Diabetes: An Online Survey
Source: Int J Environ Res Public Health. 2020 Dec 8;17(24):9180. doi: 10.3390/ijerph17249180 (PMC7764030; doi:10.3390/ijerph17249180)
Supplement: Supplementary file 1 [file ijerph-17-09180-s001.pdf]

Table S1: The Theoretical Domains Framework integrated into the COM-B model (adapted from Cane et al 2012, pg. 15) [1].

**COM-B component**		**TDF domain**
Capability	Psychological	Knowledge
		Cognitive and interpersonal skills
		Memory, attention and decision processes
		Behavioural regulation
	Physical	Physical skills
Opportunity	Social	Social influences
	Physical	Environmental context and resources
Motivation	Reflective	Social/professional role and identity
		Beliefs about capabilities
		Optimism
		Intentions
		Goals
		Beliefs about consequences
	Automatic	Reinforcement
		Emotion

Table S2: Responder comments on barriers to weight loss mapped to the Theoretical Domains Framework.

"Stress levels!! I find when I am stressed finding time to exercise and plan healthy meals becomes harder!!"<br>TDF domain: Emotion<br>COM-B component: Motivation – Automatic
"Fussy kids makes it hard to eat healthy or I need to cook 2 meals. Also stress eating from dealing with 2 under 2."<br>TDF domains: Environmental Context and Resources, Emotion, Beliefs about Capabilities<br>COM-B components: Opportunity – Physical, Motivation – Automatic, Motivation – Reflective
"I lose weight/stick to a diet better if there's is an external person/group that I need to share progress with, if I'm doing it on my own it's too easy to make excuses."<br>TDF domain: Social Influences<br>COM-B component: Opportunity: Social
"Unable to do exercise (jogging) I did pre pregnancy due to pelvic floor/back weakness."<br>TDF domain: Environmental Context and Resources, Beliefs about Capabilities<br>COM-B component – Opportunity – Physical, Motivation - Reflective
"I have been prioritising time with my boys over exercising and when I feel tired (which is almost always!) I often find myself snacking on sugary foods that I know I don't need but crave for that quick energy 'fix'."<br>TDF domains: Behavioural Regulation, Reinforcement<br>COM-B components: Capability – Psychological, Motivation - Automatic
"Having two young children and working full time I do not have the time to prep gourmet or difficult meals. It is also really important to me to sit down as a family and have dinner and I don't want to be having a shake while my family eat dinner."<br>TDF domains: Optimism, Beliefs about Consequences, Environmental Context and Resources, Social Influences<br>COM-B components: Opportunity – Social, Opportunity – Physical, Motivation – Reflective
*"I work and juggle kids and life on my own for half the year as my hubby is Fifo (*Fly in Fly Out*) so it can be difficult to find time to make healthy meals."*<br>TDF domain: Beliefs about Capabilities<br>COM-B component: Motivation – Reflective
"Looking after an 8 month old and 3 and a half year old), so a bit tired and lacking motivation. When I try to eat more healthily, I put on weight and feel hungry, so then I give up."<br>TDF domain: Beliefs about Capabilities<br>COM-B component: Motivation – Reflective
"Most revolves around time. I have an 18 month old and a 3 year old premmie with lots of extra appointments and attention its hard to stick to a diet or eating plan and when I do get a minute I'm tired. And I find it hard to spend money on myself instead of the kids too."<br>TDF domains: Environmental Context and Resources, Beliefs about Capabilities, Memory, Attention and Decision Processes<br>COM-B components: Opportunity – Physical, Capability – Psychological, Motivation – Reflective
"Taking motilium, a breastfeeding medication that helps lactation - the side effect is increased appetite. For this reason I say my family responsibility (my decision to keep breastfeeding) is taking a priority over weightloss."<br>TDF domains: Environmental Context and Resources, Beliefs about Capabilities, Beliefs about Consequences<br>COM-B components: Opportunity – Physical, Motivation - Reflective
"I would love to join a gym but don't have the funds to do so."<br>TDF domain: Environmental Context and Resources<br>COM-B model: Opportunity – Physical

“Feeding family same meal. Cost of healthy food. Time to prepare meals.”<br>TDF domain: Environmental Context and Resources<br>COM-B model: Opportunity – Physical
“There is so much conflicting information about weight loss which is confusing.”<br>TDF domain: Knowledge<br>COM-B component: Capability – Psychological
“Breastfeeding: struggling with low milk supply so worried any change to diet (particularly decreasing intake) might affect this.”<br>TDF domain: Environmental Context and Resources<br>COM-B model: Opportunity – Physical
“Results from every type of diet have been temporary, and there is overwhelming evidence this is almost always true: knowing this demotivates me.”<br>TDF domains: Knowledge, Beliefs about Capabilities, Optimism<br>COM-B models: Capability – Psychological, Motivation – Reflective
“When you have shift work and fussy eaters sometimes it is easier just to make something that the whole family will eat.”<br>TDF domain: Environmental Context and Resources<br>COM-B model: Opportunity – Physical
“Discouraged because whenever I do lose weight always put it back on very quickly & often even more besides.”<br>TDF domain: Beliefs about Capabilities<br>COM-B model: Motivation – Reflective
“With a previous history of an eating disorder I do not wish to engage in any form of restrictive dieting.”<br>TDF domain: Environmental Context and Resources<br>COM-B component: Opportunity – Physical
“Time, state of mind, lack of quick results all make it difficult for me to remain motivated.”<br>TDF domain: Beliefs about Capabilities<br>COM-B model: Motivation - Reflective
“Poly cystic ovarian syndrome”<br>TDF domain: Environmental Context and Resources<br>COM-B model: Opportunity - Physical

Table S3. Responder comments regarding opinions of the '5:2 diet'.

"I actually found it quite easy to stick to and liberating."
"It sounded like another fad diet at first but now I think it could work."
"Am currently doing the 5:2 diet and am loosing weight steadily. I find it a very achievable way to mix life and diet together without having the diet aspect impact heavily on day to day life."
"The diet was mostly successful for me. My only issue was headaches on fasting days. I eventually reached my target weight and slowly stopped following the diet and lost contact with my support group. I have since regained all of the lost weight plus some."
"I think it sounds like a positively horrific idea!"
"I am breastfeeding. I wouldn't want to drop my calories drastically for two days in a week."<br>*"It doesn't seem applicable to people with allergies to the sorts of foods that you can eat."*<br>*"Friend doing it with great success but she is very disciplined with no kids. Would love to try it but need support with menu plan so I dont have to sit and calculate kJ's."*
"I did the 5:2 diet in between having my first child and second pregnancy and found it fantastic once I got used to it. I have thought about trying again but have struggled due to being tired, busy and lacking motivation."
"Worried I'd get too hungry on the 2 days fasting and wouldn't be able to cope."
"I think I might take the 5 days and go a bit wild and eat the wrong foods. I'm not sure how I'd handle the 2 either, especially with normal eating on the other days."<br>*"The idea of it scares me a bit."*
"There is no way I could do this without husband/family thinking it was disordered."
"I tried it but had trouble working it into my life. I like the concepts but had difficulty embracing it."
"It's easy enough and gets good results but can be socially inhibiting and gets a bit tiresome."
"I worry that I wouldn't be able to stay focused at work and have energy to exercise."<br>*"I found it hard to stick to but I really liked the theory behind it I watched the doco and bought the book. I would be interested to try it again with more support."*
"I have heard of it and in principle it makes sense but I think would be hard when have to prepare meals for rest of family. Would never subject kids to it. "<br>*"I feel like it's worth a try but not sure where to start."*

References

1. Cane, J.; O'Connor, D.; Michie, S. Validation of the theoretical domains framework for use in behaviour change and implementation research. *Implement. Sci.* **2012**, *7*, 27–37.
